# Change of Vascular Endothelial Growth Factor Levels following Vitrectomy in Eyes with Proliferative Diabetic Retinopathy

**DOI:** 10.1155/2019/6764932

**Published:** 2019-10-23

**Authors:** Hui-Jin Chen, Zhi-Zhong Ma, Ying Li, Chang-Guan Wang

**Affiliations:** Department of Ophthalmology, Beijing Key Laboratory of Restoration of Damaged Ocular Nerve, Peking University Third Hospital, Beijing 100191, China

## Abstract

**Purpose:**

To study the change of concentrations of vascular endothelial growth factor (VEGF) in vitreous cavity after vitrectomy in eyes with proliferative diabetic retinopathy (PDR).

**Methods:**

In this retrospective study, intravitreal fluid samples were taken at baseline (beginning of the vitrectomy) and postoperatively (several days later after vitrectomy) at the time of prophylactic injection of bevacizumab in forty-eight eyes of forty-eight patients with PDR. Postvitrectomy fluid samples were divided into four groups according to the time interval between the vitrectomy and the injection (group 1, 3–5 days; group 2, 6–10 days; group 3, 11–15 days; group 4, 16–21 days; twelve eyes in each group). Postvitrectomy fluid sample was paired with baseline sample for each eye. VEGF concentrations in the samples were determined by enzyme-linked immunosorbent assay. Recurrent vitreous hemorrhage and neovascular glaucoma within six months postvitrectomy were also analyzed.

**Results:**

Overall, the intravitreal VEGF level after vitrectomy (median, 36.95 pg/ml; range, 3.2–1,299.4 pg/ml) was significantly less than the VEGF level at baseline (median, 704.5 pg/ml; range, 30.6–1,981.1 pg/ml). Postoperative and baseline VEGF levels were significantly correlated (*r* = 0.499, *p* < 0.01). Both the absolute value of postoperative VEGF concentrations and the postop/baseline VEGF ratios declined with time and dramatically decreased in groups 3 and 4. In only two eyes, the postoperative VEGF level was even higher than the baseline VEGF level (postop/baseline VEGF ratio >1), and recurrent vitreous hemorrhage developed within six months in these two eyes.

**Conclusions:**

After vitrectomy for PDR, intravitreal VEGF levels decreased substantially in the majority of patients, while persistent high-VEGF level occurred in a few individuals. Postoperative VEGF levels and postop/baseline VEGF ratio declined with time. The postop/preop VEGF ratio may serve as a predictor for late complications.

## 1. Introduction

Vascular endothelial growth factor (VEGF) is a potent angiogenic factor induced by retinal ischemia, and its level in vitreous fluid is significantly higher in patients with proliferative diabetic retinopathy (PDR) [1, 2], the most common cause of severe visual loss in diabetes.

Vitrectomy with membrane dissection and panretinal photocoagulation (PRP) is the standard surgical treatment for PDR patients who have nonclearing vitreous hemorrhage (VH) and progressive traction retinal detachment. However, late postoperative complications like neovascular glaucoma (NVG) and/or recurrent VH are still unavoidable following vitrectomy [3–6]. It is of interest to investigate postvitrectomy VEGF levels in PDR patients. However, very few studies have focused on this issue. Itakura et al. [7] measured VEGF levels in fluid samples obtained during fluid-air exchange 5 to 36 days after vitrectomy in 17 eyes of 15 patients with PDR. They found the VEGF levels were maintained high in the vitreous cavity postoperatively in their case series; therefore, the authors proposed that there was persistent oversecretion of VEGF into vitreous cavity even after vitrectomy for PDR. However, this study only looked at the VEGF levels in those eyes that had residual VH and needed fluid-air exchange. In addition, more than one-third of (6 of 17) the studied eyes finally developed iris rubeosis or NVG, suggesting that eyes chosen in this study may represent eyes with more postoperative complications. Similarly, VEGF levels were reported to be significantly elevated in nine PDR eyes requiring second vitrectomy due to late VH [8]. Both aforementioned studies [7, 8] have more likely chosen those patients with postoperative complications rather than the general PDR patients who underwent vitrectomy. Therefore, the purpose of this study was to evaluate whether intravitreal VEGF concentrations remain elevated after vitrectomy in more generalized patients with PDR. We also intended to investigate on the alteration of intravitreal VEGF levels with time following vitrectomy for PDR.

## 2. Patients and Methods

### 2.1. Patients

Patients with diabetes who underwent pars plana vitrectomy for PDR, between Feb 2016 and Oct 2016, at Peking University Third Hospital were retrospectively studied. The indication for vitrectomy was complications of PDR such as nonclearing VH and/or progressive traction retinal detachment. Exclusion criteria were as follows: (1) a history of prior vitreoretinal surgery except laser photocoagulation, (2) a history of intravitreal anti-VEGF drug injection before the surgery, (3) preexisting iris and chamber angle neovascularization before the surgery, (4) eyes without either baseline or postvitretomy intravitreal fluid sample like eyes filled with silicone oil or gas, and (5) eyes with less than 6 months of follow-up after primary vitrectomy.

The study was approved by the institutional review committee of Peking University Third Hospital. The research followed the tenets of the Declaration of Helsinki. Informed consent was obtained from the subjects.

## 3. Clinical Data Analysis

Demographic and clinical data from the initial examination, during vitrectomy, and follow-up visits were recorded in a database. Preoperative data included age; sex; duration and status of diabetes mellitus (fasting blood glucose, HbA1C); other systemic diseases such as hypertension; renal status (serum creatinine); medications such as anticoagulant for systemic disease; prior history of laser photocoagulation; and ophthalmic factors including best-corrected visual acuity (BCVA), lens status, fundus examination, and intraocular pressure (IOP). Intraoperative data included surgical procedures and surgical findings such as presence or absence of VH, fibrovascular proliferation (FVP), and traction retinal detachment (TRD). Postoperative data included BCVA; IOP; episodes of late complications such as recurrent VH, NVG, and residual or recurrent retinal detachment. Postoperative recurrent VH was defined as a new episode of VH occurring later than 4 weeks after primary surgery. NVG was defined as stromal and chamber angle neovascularization, with IOP elevated to 25 mm Hg or higher.

### 3.1. Surgical Technique

All patients underwent standard pars plana vitrectomy under local anesthesia. Vitrectomy was performed by two surgeons (W.C.G. or C.H.J.) using a 23-gauge 3-port system with a high-speed vitreous cutter (7500 cycles/min). Phacoemulsification was performed simultaneously in patients with cataract. Fibrovascular membrane dissection, segmentation, and delamination were performed mainly by the vitreous cutter, followed by posterior vitreous surface removal. Intraoperative bleeding was controlled either by increasing the irrigation pressure or endodiathermy. To identify rebleeding from vascular membrane or fragile vessels during vitrectomy, the IOP was decreased temporarily at the end of membrane manipulation. If the patient was taking an anticoagulant for underlying systemic diseases, anticoagulant was discontinued 1 week before surgery and resumed within 1 week after surgery. The vitreous body was removed as far as the vitreous base under scleral depression (vitreous base shaving) and blood clots in the peripheral vitreous skirt were also removed by vitreous base shaving. PRP was applied up to the ora serrata with 1 burn width apart.

### 3.2. Bevacizumab Administration

Exclusion criteria for IVB after vitrectomy include the following: (1) eyes with fibrovascular membranes in regression, (2) eyes tamponade with silicone oil, which can reduce the chance of postvitrectomy NVG and VH, and (3) patients who were over 80. Except for these exclusion criteria, all eyes received intravitreal injection of 1.25 mg/0.05 ml bevacizumab (Genentech, San Francisco, CA) after vitrectomy. The injection was not given at the end of the surgery but several days later after the surgery. The interval between vitrectomy and IVB differed among individuals, who were not randomly assigned; different timelines formed naturally. All injections were given using a standard sterilization procedure that included the use of topical povidone-iodine and levofloxacin drops. After IVB, complications such as uveitis, endophthalmitis, effects of ocular toxic effects, or any obvious systemic adverse events were observed.

### 3.3. Sample Collection and Measurements of VEGF

Samples (0.2 ml each time) were taken at baseline (beginning of the vitrectomy before intraocular infusion) and postoperatively (several days later after vitrectomy) at the time of prophylactic IVB. Samples were collected into sterile tubes and centrifuged for 10 minutes at 4°C at 3000 rpm, and the liquid component without sediment was immediately stored at −80°C until analysis. Postvitrectomy fluid samples were divided into four groups according to the time interval between the vitrectomy and the injection (group 1, 3–5 days; group 2, 6–10 days; group 3, 11–15 days; group 4, 16–21 days). For each eye, postvitrectomy fluid sample was paired with baseline sample. Both the baseline and postvitrectomy VEGF levels were measured using enzyme-linked immunosorbent assay for human VEGF (R&D Systems Inc, Minneapolis, Minnesota).

### 3.4. Statistical Analysis

Statistical analysis of the data was performed using a commercially available statistical software package (SPSS for Windows, version 19.0: SPSS, Inc, Chicago, Illinois, USA). The Kolmogorov–Smirnov test was used to test the normality of data distribution. If the data were not normally distributed, nonparametric tests were used. Difference in demographic and clinical data among the four study groups was analyzed with the chi-square or Kruskal–Wallis tests when appropriate. The Wilcoxon sum rank test was used to compare VEGF concentrations at baseline and after vitrectomy in paired samples. Differences in postoperative VEGF concentrations and postop/baseline VEGF ratio between any two groups were estimated with the Mann–Whitney *U* test. Spearman correlation was calculated to study the correlation between VEGF levels at baseline and after vitrectomy. Two-tailed probabilities of less than 0.05 were considered to indicate statistical significance.

## 4. Results

Forty-eight eyes of forty-eight patients with PDR who underwent vitrectomy were included in this study. Patient demographics and baseline ocular findings are summarized in Table 1. No significant differences were found among the four groups.

**Figure 1:**
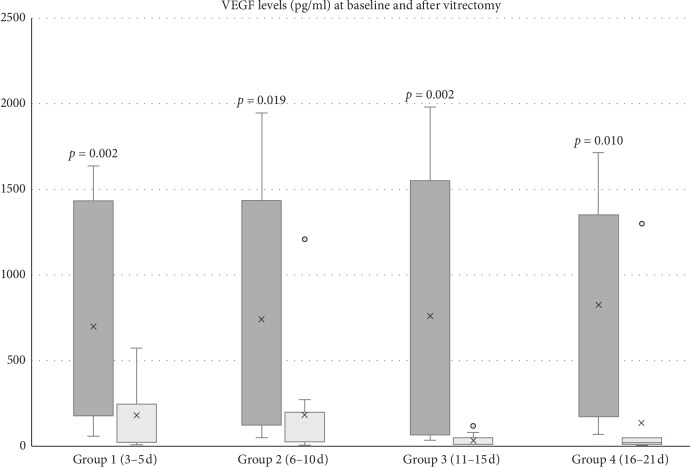
Vitreous VEGF levels at baseline and after vitrectomy. In any group, postoperative VEGF levels were significantly lower than baseline VEGF levels. This box and whisker plot shows the distribution of data into quartiles (box) with the median (dash line), the mean (cross mark), the minimum and maximum (whiskers), and the outliers (dot), which were the two eyes with even higher postoperative VEGF level than baseline.

**Figure 2:**
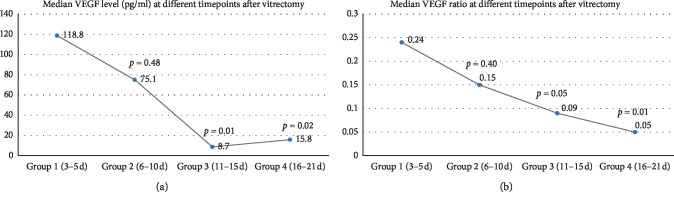
Change of postoperative VEGF levels over time. Both the median postoperative VEGF level (a) and the median postop/baseline VEGF ratio (b) were declined with time and dramatically decreased in group 3 (11–15 days) and 4 (16–21 days).

Overall, the median VEGF level in the vitreous cavity was 704.5 pg/mL (range, 30.6–1,981.1 pg/mL) at the time of vitrectomy and 36.9 pg/mL (range, 3.2–1,299.4 pg/mL) after vitrectomy. The postoperative VEGF levels decreased substantially compared with baseline VEGF levels (*p* < 0.001). A significant positive correlation was observed between the VEGF levels at baseline and after vitrectomy (*r* = 0.499, *p* < 0.001). When looking at four different groups separately, the median VEGF level after vitrectomy at each group was also significantly decreased in contrast with the VEGF level of the paired sample at baseline (Figure 1). The median postoperative VEGF level and the median postop/baseline VEGF ratio at different time intervals are shown in Figure 2. They both dramatically decreased in groups 3 and 4. When looking at each case independently, postoperative VEGF levels were lower than baseline VEGF levels in 46 out of 48 (96%) eyes, but even higher than baseline VEGF levels in two eyes (postop/preop VEGF ratio = 5.45, 1.99, respectively), in which recurrent VH developed within 6 months despite IVB injection after vitrectomy. VH occurred at 132 days after vitrectomy in one eye in group 2 and at 156 days after vitrectomy in the other eye in group 4. Retinal reattachment was achieved in all cases. None of the patients had recurrent retinal detachment or postoperative NVG. No complications were found after IVB injection. Overall, BCVA was significantly improved from 1.73 logMAR units (range, 0.4–2.6) at baseline to 0.6 logMAR units (range, 0.1–1.2) at final visit after vitrectomy (*p* < 0.001). No significant difference was found among the four groups (*p*=0.64).

## 5. Discussion

Two previous studies [7, 8] proposed intravitreal VEGF level remained high even after vitrectomy. However, these two studies selected patients who sustained more postoperative complications. It remains uncertain whether the postvitrectomy VEGF level is still high in general PDR patients.

In theory, postoperative VEGF level may be affected by the following factors: First, VEGF leftover in vitreous cavity at the end of surgery, which could be assumed undetectable since most of the VEGF in vitreous cavity should be eliminated during vitrectomy. Second, new secretion of VEGF after vitrectomy, which is associated with the ischemic level of the retina. It has been demonstrated that the VEGF level declined after PRP in PDR eyes [1]. One can speculate that new secretion of VEGF should be decreased when PRP starts to work and retina becomes quiescent after vitrectomy. Third, clearance of VEGF after vitrectomy. It has been demonstrated in the animal study that intravitreal VEGF clearance is accelerated in vitrectomized eyes [9, 10]. Therefore, it is reasonable to hypothesize that the overall VEGF level in vitreous cavity should be decreased after vitrectomy, unless VEGF is persistently overproduced due to insufficient PRP, residual retinal detachment, or other unknown reasons.

In the current study, we clearly showed that VEGF levels in the vitreous cavity decreased substantially after vitrectomy. Overall, the median postoperative VEGF level in the vitreous cavity was 704.5 pg/mL (range, 30.6–1,981.1 pg/mL); in contrast, the median baseline VEGF level in the vitreous cavity was 36.9 pg/mL (range, 3.2–1,299.4 pg/mL). In this study, we divided the postoperative samples into four groups (group 1, 3–5 days; group 2, 6–10 days; group 3, 11–15 days; group 4, 16–21 days), and the baseline data among the four groups were comparable (Table 1). When looking at each group separately, the VEGF levels after vitrectomy were also significantly decreased compared with the VEGF levels at the time of vitrectomy in all time points (Figure 1). These results support our aforementioned hypothesis. Furthermore, we found both the absolute value of postoperative VEGF concentrations and the postop/baseline VEGF ratios decreased dramatically in groups 3 (11–15 days) and 4 (16–21 days) (Figure 2). This finding is in consistency with the conception that PRP takes approximately 2∼3 weeks to function.

Previous publications have identified that the vitreous level of VEGF at the time of vitrectomy is associated with a significant risk of both the postoperative progression of PDR and the occurrence of postoperative complications [11–14]. Funatsu et al. [11] assessed the severity of diabetic retinopathy one day before and six months after vitrectomy using the ETDRS severity score. They found the vitreous levels of VEGF at the time of surgery were significantly higher in patients who showed no improvement and/or progression of PDR after vitrectomy when compared with the levels in patients who showed regression of PDR after surgery. Wang et al. [12] also showed that the increased vitreous VEGF level at the time of vitrectomy was associated with the progression of PDR after the surgery, which was defined as occurring complications like recurrent VH, FVP, NVG or TRD during follow-up over six months. Suzuki et al. [13] reported that postoperative complications developing within 24 months after PPV in PDR eyes were significantly more frequent in the high-VEGF group (≥5,000 pg/mL) than in the low-VEGF group (<5,000 pg/mL). Wakabayashi et al. [14] analyzed the relationship between the vitreous VEGF levels at the time of vitrectomy and the main postoperative complications of early VH and NVG occurring during follow-up in PDR eyes. They found the vitreous levels of VEGF at the time of surgery were significantly higher in eyes with early VH and NVG than in those without. Multivariate logistic regression analysis showed that the higher vitreous VEGF level was a significant risk factor for early VH after vitrectomy for PDR (odds ratio = 5.1); however, they found no significant relation between vitreous VEGF levels at the time of vitrectomy and postoperative late VH. In accordance with this finding, several studies [15–17] have demonstrated that anti-VEGF injection prior to vitrectomy in PDR eyes can only reduce early VH rather than the late VH after the surgery. All these results suggest that the vitreous VEGF levels at the time of vitrectomy may be closely associated with early VH after surgery and therefore may be a useful predictor of early VH. In contrast, postoperative late VH may have less association with the vitreous VEGF levels at the time of vitrectomy. This phenomenon could partly be explained by the findings showed in this study that dramatic alterations exist between the vitreous VEGF levels at the vitrectomy and after vitrectomy. Measurement of baseline VEGF levels could not substitute that of postoperative VEGF levels, although they were found significantly correlated in this study (*r* = 0.499, *p* < 0.001); therefore, it is reasonable that the vitreous VEGF levels at the vitrectomy have less predictive effect on postoperative late complications. In one previous study [8], vitreous samples were collected from nine eyes with late VH undergoing second vitrectomy. VEGF levels were found to remain high in these eyes (median, 1,610 pg/mL); therefore, the authors proposed that persistent overproduction of intraocular VEGF after vitrectomy may be associated with postoperative late VH. Interestingly, in our study, postoperative intravitreal VEGF level was found much higher than at the baseline in two individuals who subsequently had recurrent VH within six months (postop/baseline VEGF ratio = 5.45, 1.99, respectively). This finding may suggest that persistent overexpression of VEGF after vitrectomy do present in a few individuals, and the relative ratio of postoperative VEGF level to baseline VEGF level may potentially be a good predictor for late-onset complications after vitrectomy for PDR; however, further prospective study with larger number of patients is needed to prove this.

Due to the pivotal role of VEGF in the pathogenesis of PDR, numerous reports have focused on the adjunctive use of anti-VEGF agent for vitrectomy for PDR [15–25]. Most retinal surgeons perform anti-VEGF injections before vitrectomy. The main purpose of preoperative injection is to reduce intraoperative bleeding and shorten the surgical time although it can also reduce postoperative early VH (less than 4 weeks). However, the more concerned issue of PDR surgery is postoperative late complications such as recurrent late VH (more than 4 weeks) and NVG rather than intraoperative bleeding. Intraoperative bleeding can be controlled without anti-VEGF, whereas postoperative late VH and NVG are unavoidable even in the hands of the most experienced retinal surgeons. Preoperative anti-VEGF injection has little effect on lowering the late-onset VH [15–17], probably because the drug was removed at the time of vitrectomy. Besides, preoperative anti-VEGF injection may have approximately 5% of risk of membrane contraction and retinal detachment aggravation [21, 22] although it can be greatly minimized if surgery is performed few days (3–5 days) after injection. Anti-VEGF injection at the end of vitrectomy has also been attempted by some authors [23–25]. However, the intraocular VEGF level may be theoretically the lowest at the end of the surgery. When VEGF level rises again several days after vitrectomy, the drug concentration may be declined, given that the half-life of 1.25 mg intravitreally injected bevacizumab is only a few days in vitrectomized eyes in the animal model [26]. Based on all these considerations, we were more interested in the potential use of anti-VEGF application in the postoperative period (several days after vitrectomy) in PDR patients. Little has been reported regarding the postoperative use of anti-VEGF agents in the literature. In a few studies [27–29], IVB was used to manage recurrent VH after vitrectomy. In one of these studies [29], Ferenchak pointed out that patients with a history of recurrent VH after vitrectomy may also benefit from prophylactic injections at regular intervals when their vitreous cavity is clear. These studies showed the potential benefit of postoperative use of anti-VEGF agents in preventing postoperative late complications. Therefore, in this study, bevacizumab was neither given before nor at the end of vitrectomy. Instead, it was given after the vitrectomy with a short interval between the surgery and the injection. We did not restrict the time interval on purpose because there is no definite answer regarding the best time after surgery to perform the injection. The varied time intervals formed naturally. We found that the majority of the patients (96%) did not develop recurrent VH and none of the patients had postoperative NVG within 6 months after vitrectomy. It is reasonable to suppose that postoperative prophylactic IVB may reduce or postpone these complications. However, further randomized controlled study with larger number of patients and longer period time of follow-up is needed to draw this conclusion. In addition, in two eyes with high postop/preop VEGF ratio (>1), VH reoccurred even after postoperative prophylactic IVB. It could be partly explained by the well-documented fact that the pharmacokinetics of intravitreal drugs are faster in vitrectomized eyes [10, 26, 30–32]. Therefore, whether increased dosing or more frequent injections postvitrectomy should be considered in high-risk eyes (postop/preop VEGF ratio >1) also needs to be elucidated in future study.

There were some limitations to this study. First, this study was retrospective in nature. We did not randomly assign the eyes into four groups according to different time intervals. The varied time intervals formed naturally. Although the baseline data among the four groups were comparable, there could be some unpredictable factors influencing our result. Second, in this study, we only measured the postoperative intravitreal VEGF levels in eyes filled with BSS, and eyes filled with silicone oil or gas might have higher postoperative VEGF level, but this was not addressed by the current study. Therefore, the possibility of selection bias with respect to severity of the disease has to be considered in interpreting the results of this study. Third, there was no conclusive answer in this study whether postoperative prophylactic anti-VEGF therapy can reduce the postoperative late complications; therefore, a randomized controlled study with larger number of patients was needed to further address this issue. Nevertheless, our study was the first study to compare the difference between VEGF levels at the time of vitrectomy and after vitrectomy in PDR patients and analyze the change of postoperative VEGF level with time. Our study was also one of the few studies to explore prophylactic anti-VEGF therapy in the postoperative period. The findings of this study may help to develop greater insight into the pathophysiology resulting in postoperative late-onset complications in PDR and establish new options for future intervention in these patients.

In conclusion, VEGF levels decreased substantially after vitrectomy in the majority of PDR patients, although postoperative VEGF levels were significantly correlated with VEGF levels at the vitrectomy. In general, both the absolute value of postoperative VEGF concentrations and the postop/baseline VEGF ratio decreased with time. However, persistent overexpression of VEGF after vitrectomy occurred in a few individuals and may be associated with late complications after surgery. In these high-risk eyes (postop/preop VEGF ratio >1), repeat prophylactic anti-VEGF therapy after vitrectomy may be reasonable to prevent postoperative late complications, although further studies are needed to prove this.

## Figures and Tables

**Table 1:** Patient demographics and baseline ocular findings.

Characteristic	No. (%) or median (range)	*p* value
Sex: male/female	22/26 (45.8/54.2)	0.80
Age (y)	57 (37–75)	0.39
Duration of diabetes (y)	14 (3–33)	0.97
Fasting blood glucose (mmol/L)	6.9 (5–8.5)	0.52
Hb^*∗*^A1c (%)	7.5 (5.4–10.3)	0.40
Hypertension	32 (66.7)	0.86
Serum creatinine (mg/dL)	0.89 (0.27–9.2)	0.74
Oral anticoagulant	19 (39.6)	0.81
Preoperative BCVA (logMAR^†^)	1.73 (0.4–2.6)	0.72
Preoperative IOP (mmHg)	15 (11–22)	0.88
Cataract	15 (31.2)	0.43
Pseudophakia	10 (20.8)	0.22
Previous PRP^‡^	20 (41.7)	0.87
Vitreous hemorrhage	44 (91.7)	0.54
Fibrovascular proliferation	43 (89.6)	0.48
Traction retinal detachment	23 (47.9)	0.82

^*∗*^Hb, hemoglobin; ^†^MAR, logarithm of minimal angle of resolution; ^‡^PRP, panretinal photocoagulation; *p* > 0.05 in this table means no statistically significant differences were found among the four groups.

## Data Availability

The data used to support the findings of this study are available from the corresponding author upon request.
